# Serum Concentrations of Fibroblast Growth Factors 19 and 21 in Women with Gestational Diabetes Mellitus: Association with Insulin Resistance, Adiponectin, and Polycystic Ovary Syndrome History

**DOI:** 10.1371/journal.pone.0081190

**Published:** 2013-11-19

**Authors:** Dongyu Wang, Wenjing Zhu, Jieming Li, Chongyou An, Zilian Wang

**Affiliations:** Department of Obstetrics and Gynecology, The First Affiliated Hospital of Sun Yat-sen University, Guangzhou, Guangdong, China; University of Louisville School of Medicine, United States of America

## Abstract

**Background:**

Fibroblast growth factor 19 (FGF19) and FGF21 are considered to be novel adipokines that improve glucose tolerance and insulin sensitivity. In the current study, we investigated serum FGF19 and FGF21 levels in patients with gestational diabetes mellitus (GDM) and explored their relationships with anthropometric and endocrine parameters.

**Method:**

Serum FGF19 and FGF21 levels were determined by enzyme-linked immunosorbent assay (ELISA) in patients with GDM (n = 30) and healthy pregnant controls (n = 60) matched for maternal and gestational age. Serum FGF19 and FGF21 levels were correlated with anthropometric, metabolic, and endocrine parameters.

**Results:**

Circulating levels of FGF19 were significantly reduced in patients with GDM relative to healthy pregnant subjects, whereas FGF21 levels were increased in GDM patients. Serum FGF19 levels independently and inversely correlated with insulin resistance (increased homeostasis model assessment of insulin resistance, HOMA-IR) and were positively related to serum adiponectin in both groups. In contrast, serum FGF21 levels independently and positively correlated with insulin resistance and serum triglycerides and were inversely related to serum adiponectin. In addition, in the combined population of both groups, those women with preconception polycystic ovary syndrome (PCOS) history had the lowest levels of FGF19, which were significantly lower than those in GDM patients without PCOS history and those in controls without PCOS history.

**Conclusions:**

Circulating FGF19 levels are reduced in GDM patients, in contrast with FGF21 levels. Both serum FGF19 and FGF21 levels are strongly related to insulin resistance and serum levels of adiponectin. Considering the different situation between FGF19 and FGF21, we suggest that reduced serum FGF19 levels could be involved in the pathophysiology of GDM, while increased serum FGF21 levels could be in a compensatory response to this disease.

## Introduction

In the past decade, there has been a significant increase in the incidence of gestational diabetes mellitus (GDM) [1]. Risks of adverse maternal and fetal outcomes are increased as a consequence of GDM [2]. GDM poses an increased risk for development of type 2 diabetes mellitus (T2DM), obesity, and even cardiovascular diseases later in life for both mother and child [3]–[5]. Hyperglycemia alone does not account for all the complications of GDM, because GDM pregnancies that are under optimal control of maternal blood glucose levels can still be prone to the same gestational and postpartum complications [6].

Insulin resistance, the pancreatic β-cell dysfunction, and lipid metabolism disorders may contribute to the development of GDM and GDM associated complications. In recent years, investigators emphasize the role of cytokines and especially adipocytokines that influence insulin sensitivity as biomarkers of early impaired glucose metabolism and insulin resistance [7]. Lower adiponectin concentrations in early pregnancy predicted GDM and were positively associated with β-cell dysfunction [8], [9]. Additionally, several studies have described dysregulation of leptin [10], visfatin [11], TNF-α [12], and retinol binding protein-4 [13] in GDM. Those adipokines may involve in pathophysiology of GDM and its complications.

Fibroblast growth factor 19 (FGF19) and 21 (FGF21) belong to the “endocrine” sub-group of the FGF superfamily [14]. Recently, both factors were found to act on multiple tissues (including adipose tissue) to coordinate carbohydrate and lipid metabolism in response to nutritional status. Whereas FGF19 was mainly secreted from the small intestine in response to feeding and has insulin-like actions, FGF21 was mainly secreted from the liver in response to extended fasting and has glucagon-like effects [14]. Both factors stimulated glucose uptake in 3T3-L1 adipocytes [15]. Furthermore, comparable efficacy of both FGF19 and FGF21 was found to correct body weight and serum glucose in obese and diabetic rodents and primates [15]–[17]. In addition to its role in bile acid homeostasis, FGF19 activated an insulin-independent endocrine pathway that regulates hepatic protein and glycogen metabolism without affecting lipogenesis [18]. Additionally, FGF19 repressed gluconeogenesis in liver. On the other hand, a prominent feature of the pharmacological studies with FGF21 was its profound effect on insulin sensitivity. FGF21 improved pancreatic β-cell function and survival by activation of p44/42 mitogen-activated protein kinase [19]. Interestingly, serum FGF19 levels were lower in T2DM patients with metabolic syndrome and in obese patients [20], [21]. In contrast, increased concentrations of serum FGF21 have been found in subjects with T2DM and in obese children and adults [22], [23]. Moreover, in two 5 years follow-up studies, high levels of FGF21 predicted impaired glucose metabolism and T2DM [24], [25]. Dysregulation of FGF19 and/or FGF21 may contribute to the development of those metabolic diseases. Alternatively, the altered expression of FGF19 and/or FGF21 may be involved in a complex adaptive response to these diseases, or a phenomenon reminiscent of hyperinsulinemia and insulin resistance.

In contrast to other adipokines, few data on circulating FGF19 or FGF21 concentrations is available in GDM. Only one trial evaluating serum FGF21 concentrations in GDM showed FGF21 was independently associated with markers of insulin resistance and an adverse lipid profile in pregnancy [26]. To our best knowledge, no targeted information about the circulating FGF19 levels in GDM is available. In the present study we investigated both serum FGF19 and FGF21 concentrations in patients with GDM in one trial. In addition, we analyzed the association of serum FGF19 and FGF21 with markers of insulin resistance, glucose and lipid metabolism, as well as other adipokines.

## Subjects and Methods

### Ethics statement

The study was approved by the research ethical committee of The First Affiliated Hospital of Sun Yat-sen University and all subjects gave written informed consent before taking part in the study.

### Subjects and clinical data

This analysis was conducted as part of an ongoing cohort study in which pregnant women were recruited at the time of antepartum screening for GDM. Only ethnically Chinese women with a singleton pregnancy were included in the study. Overall, 30 pregnant women with GDM and 60 pregnant control women matched for maternal and gestational age were enrolled in the study.

The diagnosis of GDM was made when any of the following plasma glucose values were met or exceeded during a 75-g, 2-hour oral glucose tolerance test (OGTT) at 24–28^th^ weeks of gestation: fasting, 5.1 mmol/L, 1 hour, 10.0 mmol/L, and 2 hours, 8.5 mmol/L, according to the criteria established by the Ministry of Health (MOH) China [27]. Before the diagnostic 75-g OGTT was done, a fasting plasma glucose (FPG) test had been performed at the first prenatal visit in the first trimester to exclude previously undiagnosed preexisting DM (≥7.00 mmol/L).

In all subjects the following informations were available: age; clinical history (including preconception polycystic ovary syndrome PCOS history); blood pressure (systolic and diastolic blood pressure); body mass index (BMI). All subjects were between 18 and 35 years old. The diagnosis of PCOS was based on the Rotterdam consensus criteria [28]. BMI was calculated as weight in kg before pregnancy divided by height in m squared and ranged from 16.18 to 30.61 kg/m^2^ in all the study population. Patients with overt diabetes, hypertension, renal and cardiovascular diseases, preeclampsia, or any other complications were excluded from the study. All patients with PCOS history received ovulation induction treatment prior to pregnancy except three patients (two in the GDM group and one in the non-GDM group) who ovulated spontaneously after stopping oral contraceptive pills. Type and duration of induction treatment were not influenced by study participation. All patients did not take any medication after spontaneous conception.

### Methods

#### Blood sampling

Blood samples were withdrawn from an antecubital vein between 08:00 and 09:00 AM after overnight fasting at the time of OGTT. Blood samples were separated by centrifugation for 15 minutes at 1000× g after clotting for 30 minutes at room temperature. Serum samples were subsequently stored in aliquots at −80°C until further analysis of FGF19, FGF21 and adiponectin levels.

#### Hormonal and biochemical assays

Serum FGF19 levels were measured with a sandwich enzyme-linked immunosorbent assay (ELISA) (FGF19 Quantikine® ELISA kit, Cat. No. DF1900; R&D Systems, Minneapolis, MN, USA), following the manufacturer's instructions. The standard curve range for the assay was 15.6–1000 pg/ml. Serum FGF21 levels were measured by means of ELISA (FGF21 Quantikine® ELISA kit, Cat. No. DF2100; R&D Systems, Minneapolis, MN, USA), following the manufacture's instructions. The standard curve range for the assay was 31.3–2000 pg/ml. Serum adiponectin levels were measured with commercial ELISA kit (RayBio Human Adiponectin/Acrp30 ELISA Kit, USA). The standard curve range for the assay was 24.69–18000 pg/ml. All of the above serum samples were assayed in duplicate, and the mean value of the two measures was used for the analyses.

Biochemical parameters including insulin, glucose, total, low-density lipoprotein (LDL), and high-density lipoprotein (HDL) cholesterol, triglycerides, creatinine were measured in the Department of Biochemistry of The First Affiliated Hospital of Sun Yat-sen University, Guangzhou by standard laboratory methods. Homeostasis model assessment (HOMA-IR) index was calculated as previously described [29] using the following formula: fasting serum insulin (mIU/l) × fasting serum glucose (mmol/l)/22.5.

### Statistical analysis

All statistical analyses were performed with the Statistical Package for Social Science version 13.0 (SPSS, Inc., Chicago, IL). Data are expressed as mean ± standard deviations for normally distributed variables, median with interquartile range for skewed data, and as frequencies for categorical variables. Differences between groups were assessed by Student's unpaired t test, Mann-Whitney U test, or Chi-square test as appropriate. Correlation analysis was performed using the Spearman rank correlation method. To identify independent relationships and adjust the effects of covariates, multiple linear regression analyses were performed including all parameters with highly significant correlations in the univariate analysis (P≤0.01) as covariates. In case of parameters strongly related to each other, one representative covariate was included in the model. HOMA-IR was a significantly better representative covariate compared with fasting glucose or fasting insulin. Distribution was tested for normality using Kolmogorov-Smimov test, and nonnormally distributed parameters were logarithmically transformed before multiple analysis. P<0.05 was considered statistically significant.

## Results

The clinical baseline characteristics of the subgroups studied (control, GDM) are summarized in Table 1. Because subjects were matched for maternal and gestational age, both parameters were similar between the two groups. In addition, there were no statistically significant differences between GDM patients and controls with respect to blood pressure, measures of lipid metabolism (TG, cholesterol) and renal function (creatinine). In contrast, fasting plasma glucose, 1- and 2-hour glucose values during 75-g OGTT, fasting insulin, and HOMA-IR were significantly higher in GDM patients as compared with controls (P<0.001), whereas serum adiponectin levels were significantly lower in subjects with GDM (P<0.001). Furthermore, the prevalence of preconception PCOS history was significantly higher in patients with GDM than in controls (P = 0.018). Body mass index was ranged from 16.62 to 29.16 kg/m^2^ in the GDM population, 16.18 to 30.61 kg/m^2^ in the control population. There was a small but statistically significant increase in preconception BMI in GDM patients (P = 0.045).

**Table 1:** General characteristics of the study participants.

Characteristics	GDM group(n = 30)	Control group(n = 60)	P value	PCOS group(n = 12)
Age (years)	29.33±3.29	28.95±3.14	0.592	29.37±3.11
Gestational age at OGTT (weeks)	25.37±1.25	25.23±1.05	0.595	25.57±1.16
Body mass index before pregnancy (kg/m^2^)	21.80±3.03	20.36±3.37	0.045	21.73±3.06
PCOS before pregnancy (yes/no)	8/22	4/56	0.018	–
Systolic blood pressure (mm Hg)	118±13	113±17	0.623	117±11
Diastolic blood pressure (mm Hg)	73±11	68±14	0.597	72±12
Glucose 0 h (mmol/L)	5.01±0.64	4.24±0.32	<0.01	4.86±0.44
Glucose 1 h (mmol/L)	10.13±1.27	7.23±1.34	<0.01	9.31±1.22
Glucose 2 h (mmol/L)	8.70±0.96	6.35±1.01	<0.01	7.90±1.45
Fasting insulin (µU/ml)	13.07±4.20	9.21±2.94	<0.01	13.28±3.87
HOMA-IR	2.87 (2.08–3.49)	1.74 (1.36–2.10)	<0.01	2.89 (2.08–3.51)
Creatinine (µmol/L)	43±11	41±13	0.913	41±13
Triglycerides (mmol/L)	2.07±0.53	1.93±0.62	0.306	2.03±0.63
Total cholesterol (mmol/L)	6.48±1.01	6.16±0.86	0.122	6.47±0.97
HDL cholesterol (mmol/L)	2.00±0.30	1.91±0.36	0.216	1.88±0.32
LDL cholesterol (mmol/L)	3.57±0.80	3.42±0.70	0.381	3.56±0.77
Adiponectin (µg/ml)	13.58±7.21	24.24±7.44	<0.01	16.37±7.37
FGF19 (pg/ml)	65.96 (51.76–90.11)	112.62 (93.35–146.83)	<0.01	53.97 (35.80–70.80)
FGF21 (pg/ml)	124.47 (82.72–189.50)	68.10 (49.23–97.50)	<0.01	109.73 (55.96–174.42)

Data are presented as means and SD, counts, or medians and interquartile ranges, as appropriate. HOMA-IR: homeostasis model of insulin resistance; HDL: high-density lipoprotein; LDL: low-density lipoprotein. *P* values are presented for comparison between GDM subjects and control subjects. The characteristics of the PCOS group are shown.

**Figure 1:**
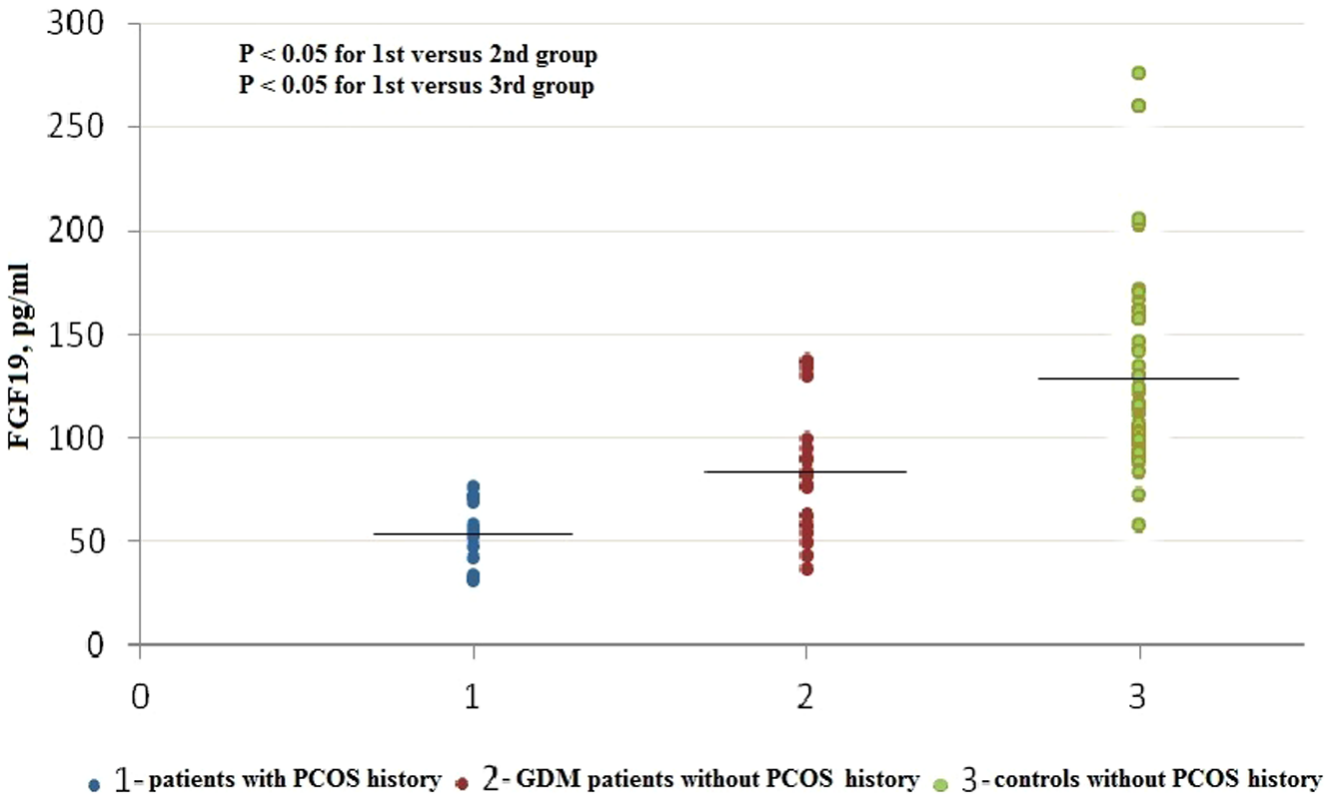
Scattergram of serum FGF19 levels in patients with PCOS history, GDM patients without PCOS history. Horizontal lines across the scatter diagram represent median values. Differences between groups were assessed by Mann-Whitney *U* test with Bonferroni adjustment for multiple testing.

**Figure 2:**
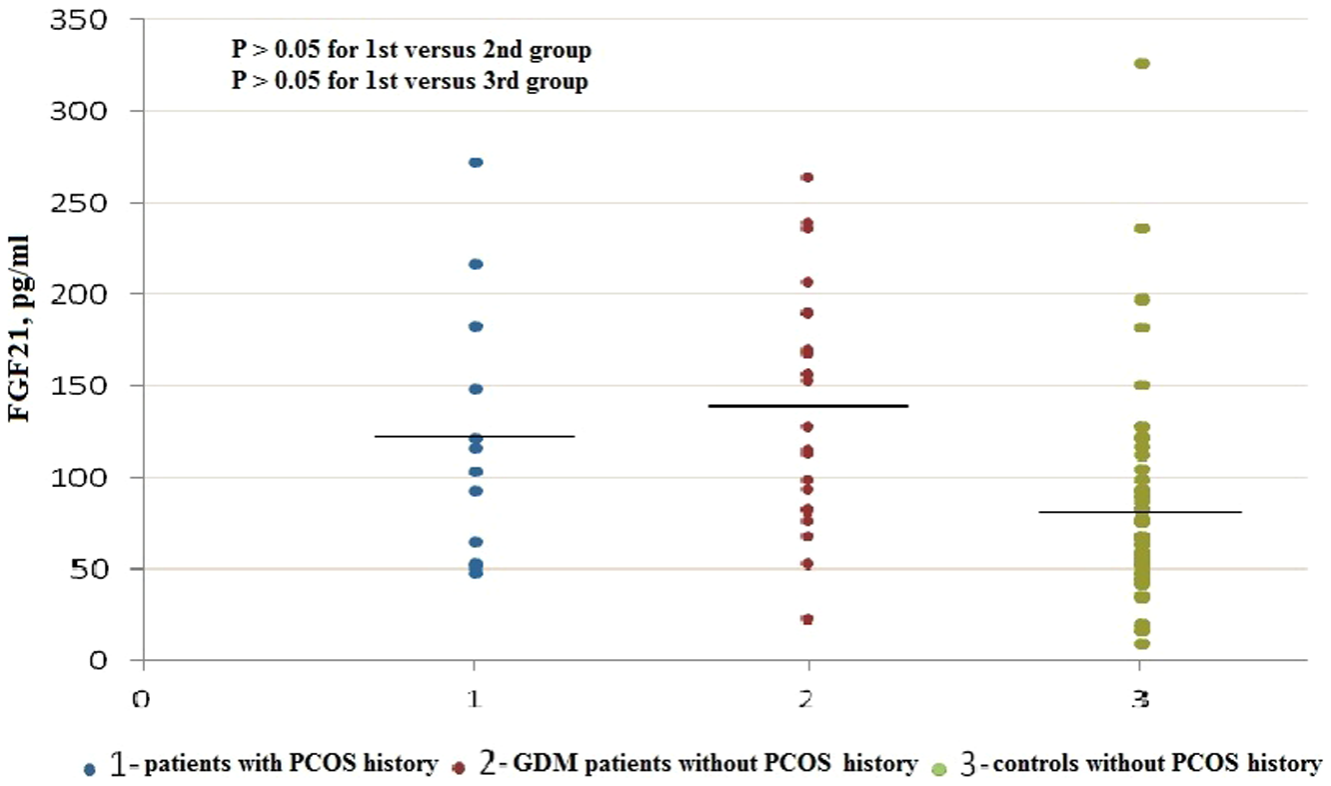
Scattergram of serum FGF21 levels in patients with PCOS history, GDM patients without PCOS history. Horizontal lines across the scatter diagram represent median values. Differences between groups were assessed by Mann-Whitney *U* test with Bonferroni adjustment for multiple testing.

Serum FGF19 levels were significantly lower in patients with GDM (median: 65.96 pg/ml; interquartile range: 51.76–90.11 pg/ml) than those in controls (median: 112.62 pg/ml; interquartile range: 93.35–146.83 pg/ml; P<0.001). In addition, in the combined population of GDM patients and controls, those women with PCOS history had the lowest levels of FGF19 (n = 12, median: 53.97 pg/ml; interquartile range: 35.80–70.80 pg/ml), which were significantly lower than those in GDM patients without PCOS history (n = 22, median: 82.73 pg/ml; interquartile range: 57.87–95.98 pg/ml, P<0.05) and those in controls without PCOS history (n = 56, median: 116.57 pg/ml; interquartile range: 95.34–151.22 pg/ml, P<0.05, Fig. 1). On the contrary, serum FGF21 levels were significantly higher in patients with GDM (median: 124.47 pg/ml; interquartile range: 82.72–189.50 pg/ml) than those in controls (median: 68.10 pg/ml; interquartile range: 49.23–97.50 pg/ml; P<0.001). Moreover, serum FGF21 levels were not significantly different in subjects with PCOS history (n = 12, median: 109.73 pg/ml; interquartile range: 55.96–174.42 pg/ml) as compared with GDM patients without PCOS history (n = 22, median: 140.41 pg/ml; interquartile range: 82.72–189.50 pg/ml, P>0.05) or controls without PCOS history (n = 56, median: 68.09 pg/ml; interquartile range: 48.38–93.20 pg/ml, P>0.05, Fig 2). First, the differences of FGF19 and FGF21 levels between GDM vs. control group were assessed by Mann-Whitney U test because both serum FGF19 and FGF21 levels in our study were nonnormally distributed parameters. Then, because BMI is an important predictor of both FGF21 and FGF19 levels in human subjects and preconception BMI was different between the groups in our study. Therefore, after log transformation was performed for FGF19 and FGF21, both parameters were compared between women with GDM and those without GDM by analysis of variance with adjustment for BMI. In specific, data comparisons between the groups were performed with a univariate general linear model and preconception BMI was chosen as a covariate. Similar results were found. The difference in both FGF19 and FGF21 levels between the groups remained significant after statistical correction for BMI (P<0.001).

We then investigated the univariate correlation coefficients of serum FGF19 and FGF21 with clinical, anthropometric and biochemical (including adiponectin levels) parameters in the combined population of both groups (Table 2). Serum FGF19 levels were significantly and negatively associated with fasting, 1-hour glucose, fasting insulin, HOMA-IR, and triglycerides. Furthermore, serum FGF19 levels correlated positively with adiponectin. Whereas serum FGF21 levels positively correlated with fasting glucose, fasting insulin, HOMA-IR, and triglycerides. In contrast, adiponectin was found to be negatively associated with circulating FGF21 levels. The above mentioned relationships were calculated also in each group separately (data not shown) and were found similar to those of the combined population. Unexpectedly, we found no significant correlation of serum FGF19 or FGF21 levels with preconception BMI. In both GDM and control groups, there was a moderate but not significant increase in serum FGF19 levels in lean (BMI<25 kg/m^2^) subjects compared with overweight (BMI≥25 kg/m^2^) subjects, while there was also a moderate but not significant decrease in serum FGF21 levels in lean subjects compared with overweight subjects. In fact, when the GDM patient population and controls were considered together, there were no statistically significant differences in serum FGF19 or FGF21 levels between lean (n = 72, FGF19: 100.16 pg/ml; FGF21: 79.56 pg/ml; BMI<25 kg/m^2^) and overweight (n = 18, FGF19: 93.10 pg/ml; FGF21: 84.66 pg/ml; BMI≥25 kg/m^2^, P>0.05) subjects. We also found no significant relationship between FGF19 and FGF21 (r = −0.162, P = 0.140).

**Table 2:** Relationships of FGF19 and FGF21 with clinical, anthropometric and biochemical parameters calculated in a combined population of healthy pregnant controls and patients with GDM (n = 90).

	Serum FGF19		Serum FGF21	
	r	P	r	P
Age (years)	−0.072	0.498	0.011	0.921
Gestational age at OGTT (weeks)	−0.056	0.599	−0.007	0.945
Body mass index before pregnancy (kg/m^2^)	−0.157	0.138	0.206	0.052
Systolic blood pressure (mm Hg)	−0.165	0.120	0.192	0.070
Diastolic blood pressure (mm Hg)	−0.021	0.844	0.158	0.138
Glucose 0 h (mmol/L)	−0.383	<0.001*	0.274	0.009*
Glucose 1 h (mmol/L)	−0.242	0.022*	0.190	0.073
Glucose 2 h (mmol/L)	−0.201	0.054	0.184	0.082
Fasting insulin (µU/ml)	−0.351	<0.001*	0.259	0.014*
HOMA-IR	−0.413	<0.001*	0.289	0.006*
Creatinine (µmol/L)	0.041	0.661	0.132	0.144
Triglycerides (mmol/L)	−0.227	0.031*	0.395	<0.001*
Total cholesterol (mmol/L)	0.095	0.373	−0.108	0.313
HDL cholesterol (mmol/L)	0.096	0.367	−0.063	0.555
LDL cholesterol (mmol/L)	0.050	0.642	−0.010	0.926
Adiponectin (µg/ml)	0.431	<0.001*	−0.377	<0.001*

The correlations were calculated in the combined population of GDM patients and controls. Units and abbreviations are as Table 1.

*Significant correlation as assessed by Spearman correlation method.

In multiple stepwise linear regression analysis, adiponectin, HOMA-IR, and PCOS history remained independently associated with FGF19 circulating levels after adjustment for age and preconception BMI. On the other hand, adiponectin, HOMA-IR, and triglycerides remained independently associated with FGF21 circulating levels after adjustment for age and preconception BMI (Table 3). Here, PCOS history was also included in the multiple analysis for FGF21, but there was no independent association between FGF21 and PCOS history after adjustment for age and preconception BMI.

**Table 3:** Multiple linear regression analysis between FGF19 (dependent variable) and age, BMI, HOMA-IR, adiponectin, and PCOS history, as well as between FGF21 (dependent variable) and age, BMI, HOMA-IR, adiponectin and TG.

	Serum FGF19		Serum FGF21	
	β	P	β	P
Age (years)	−0.054	0.544	0.098	0.337
Body mass index before pregnancy (kg/m^2^)	−0.013	0.888	0.088	0.383
PCOS history before pregnancy	−0.205	0.045*	–	–
HOMA-IR	−0.246	0.020*	0.220	0.039*
Triglycerides (mmol/L)	–	–	0.294	0.005*
Adiponectin (µg/ml)	0.343	0.001*	−0.225	0.033*

β Coefficients and P values are given. Abbreviations are indicated in Table 1.

*Significant correlation.

## Discussion

In the current study, circulating levels of FGF19 are determined for the first time in pregnant patients, to our best knowledge. We showed that serum FGF19 levels were significantly decreased in GDM patients as compared with healthy pregnant controls who were matched for maternal and gestational age. Additionally, circulating FGF19 concentrations independently and inversely correlated with insulin resistance (increased HOMA-IR, and decreased adiponectin) both in univariate correlation and multiple linear regression analysis. These findings are consistent with the proposed role of FGF19 in promoting insulin sensitivity and glucose uptake in target tissues based on experimental animal and in vitro studies [15], [30]. However, in humans, in vivo data concerning the relation of FGF19 to metabolic parameters are paradoxical. Schreuder et al. [31] did not find any influence of insulin resistance as assessed by HOMA index on intestinal FGF19 production in patients with non-alcohol fatty liver disease (NAFLD). Mráz et al. [21] also confirmed that FGF19 were not significantly related to serum glucose, insulin, or HOMA index in any of the obese, T2DM, and healthy female groups. In contrast, Reiche et al. [32] reported fasting glucose negatively and independently predicted circulating FGF19 in healthy subjects. Gallego et al. [33] further demonstrated serum FGF19 levels were inversely correlated with insulin resistance and insulin levels in HIV-1-infected patients and healthy controls. Interestingly, both Reiche and Mráz found a positive correlation between FGF19 and adiponectin [21], [32], in agreement with our finding. Here, the key conflicting problem is the relationship between FGF19 and insulin. Recent studies revealed that FGF19 shared some of the metabolic actions of insulin, namely, the stimulation of hepatic protein synthesis and glycogen synthesis, and inhibition of gluconeogenesis [18]. However, there were important differences. First, FGF19 was presumably released mainly from the small intestine, not pancreas. Second, the insulin-like effects of FGF19 were mediated by signaling pathways distinct from those employed by insulin [18]. It has been reported that in human hepatocarcinoma HepG2 cells that express FGF receptor 4 (FGFR4) and β-Klotho (co-receptor), FGF19 may utilize a FGF substrate 2α (FRS2α)-small guanosine triphosphatase (Ras)-extracellular signal-regulated protein kinase (ERK)-p90 ribosomal S6 kinase (p90RSK) pathway to induce phosphorylation of ribosomal protein S6 (rpS6) and eukaryotic initiation factor 4B (eIF4B), which improves the efficiency of global protein synthesis. Furthermore, FGF19 may act through the insulin-independent Ras-ERK-p90RSK pathway to induce phosphorylation of glycogen synthase kinase 3 (GSK3) kinases, which prevents inhibition of GS and thus increases glycogen synthesis. As is well-known, insulin utilizes the PI3K-Akt-mTOR signaling pathway to improve hepatic protein and glycogen synthesis. Unlike insulin, FGF19 did not increase hepatic triglycerides or induce sterol regulatory element-binding protein, isoform 1c (SREBP-1c)-dependent lipogenic gene expression, which requires the PI3K-Akt-mTOR signaling pathway. These findings were based on the in vitro and cellular experiments. Therefore, the exact signaling pathways activated by FGF19 in human in vivo warrant future investigation. However, FGF19 did not stimulate lipogenesis, which was a key advantage in considering the FGF19 pathway as an anti-diabetic therapy. Finally, FGF19 and insulin were temporal different. Whereas insulin was released within minutes of a meal, FGF19 serum levels peaked 2 h after a meal [34], and, accordingly, circulating FGF19 levels in humans inversely correlated with fasting glucose levels and metabolic syndrome [20], [32], [35]. On the other hand, it was demonstrated that the mouse FGF19 orthologue, FGF15, and insulin acted in liver partially via the same pathways, with forkhead transcriptional factor 1 (FoxO1), a factor controlling the expression of CYP7A1 (the first and rate-limiting enzyme in the major bile acid synthesis pathway) as well as several gluconeogenetic genes, being the key converging node [36]. Taken together, FGF19 has unique and insulin-like metabolic actions and a post-prandial hormonal program may exist in which insulin and FGF19 coordinately govern nutrient metabolism. Whereas most of the effects of FGF19 and insulin are mediated by different signaling pathways, both of the two homones may be partially influenced by each other through a converging node in the same pathways. Mráz et al. [21] did report that acute hyperinsulinemia tended to reduce FGF19 levels. Moreover, FGF19 has been reported to have glucose- and insulin-lowering actions in diabetic rodents [16]. Thus it is speculated that both FGF19 and insulin may have an inverse effect on each other. Therefore, in GDM, a highly insulin-resistant state leading to high levels of circulating insulin may tend to result in low levels of FGF19. The precise mechanism was not elucidated, and this should be an important question for further exploration. In addition, although the cross-sectional nature of the present study does not allow cause-to-effect relationships, it may be speculated that the abnormally low levels of FGF19 in GDM patients contribute to metabolic disturbances such as hyperglycemia and insulin resistance that occur commonly in these patients. Furthermore, low levels of FGF19 may contribute to the development of GDM associated long-term complications. It needs to be emphasized in this context that besides insulin, the effects of FGF19 on glucose metabolism may at least in part mediated by adiponectin, an important insulin-sensitizing and vasoprotective adipokine positively associated with pancreatic β-cell function. In this study, adiponectin remains an independent positive predictor of circulating FGF19. Alternatively, the positive correlation between FGF19 and adiponectin may reflect the fact that both are negatively regulated by insulin resistance.

Another interesting finding of the present study is that serum levels of FGF19 were significantly lower in patients with PCOS history than those without PCOS history. A potential explanation of the link between reduced FGF19 levels and PCOS history is the connection of insulin resistance with PCOS. It has been well established that hyperinsulinemia and insulin resistance were common biochemical features of PCOS independent of obesity [37]. PCOS women were at substantial risks for development of glucose intolerance, diabetes, lipid abnormalities and cardiovascular abnormalities since they often had insulin resistance. In fact, pregnant women with PCOS were more than three times as likely to develop GDM when compared with pregnant women without PCOS [38]. In accordance with these findings, our study also showed that GDM was strongly associated with PCOS history. Moreover, according to one meta-analysis [39], adiponectin levels were lower in PCOS women compared with healthy control subjects of a similar BMI. Considering that PCOS is associated with insulin resistance and adiponectin, and our finding that both insulin resistance and adiponetin were significantly associated with serum FGF19 levels, it is reasonable to explain the association of FGF19 with PCOS. However, PCOS history persisted independently associated with FGF19 circulating levels in multiple stepwise regression analysis. Besides insulin resistance and dyslipidemia, whether hyperandrogenism or dysregulations of any other hormones commonly existed in patients with PCOS might influence the serum FGF19 levels need to be further investigated. Here, we are aware that the PCOS results are based on very small numbers. This is the limitation in our current study. Larger cohort studies are needed to provide further reliable results about the relationship between FGF19 and PCOS.

Our study also evaluated the serum FGF21 levels in GDM patients. FGF21 serum concentrations showed a significant and positive association with insulin resistance and dyslipidemia including increased HOMA-IR, decreased adiponectin, and increased TG both in univariate and multivariate analysis. These findings are in accordance with the previous study in which FGF21 serum concentrations were determined in 40 patients with GDM and 80 healthy pregnant controls [26]. And it was found that serum FGF21 levels were positively associated with fasting insulin, HOMA-IR, and TG, whereas negative correlations existed with HDL and adiponectin, similar to our data. However, Stein and coworkers found serum FGF21 concentrations were not significantly different in subjects with GDM as compared with controls. For in their study, GDM patients and controls were matched for fasting insulin. In our study, GDM patients and controls were only matched for maternal and gestational age. As expected, serum levels of FGF21 were significantly higher in GDM patients as compared with controls. Obviously, this cross-sectional study can not indicate serum FGF21 concentrations as a predictor for the development of GDM. But it is worth mentioning that, in recent years, considerable data have revealed FGF21 was independently correlated with metabolic disturbances. High levels of FGF21 predicted impaired glucose metabolism (OR = 2.2; 95%CI 1.3–3.6; P = 0.002), metabolic syndrome (OR = 2.6; 95%CI 1.5–4.5; P = 0.001) and T2DM (OR = 2.4; 95%CI 1.2–4.7; P = 0.01) after adjustment for age, sex and BMI [25]. To date, the physiological significance for increased FGF21 in metabolic disease is not elucidated. Paradoxical upregulation of FGF21 might be a compensatory mechanism to improve glucose metabolism when insulin resistance and an adverse lipid profile are present [22]. Alternatively, the metabolic syndrome might cause resistance to FGF21 leading to compensatory upregulation of this antidiabetic adipocytokine as proposed by Fisher et al. [17]. Our finding is in good agreement with this hypothesis. Nevertheless, systemic administration of FGF21 markedly reduced blood glucose and insulin levels in both the diabetic rodents and primates [40]. Furthermore, FGF-21 provided sustainable glucose control without incidence of hypoglycemia and a substantial improvement in lipid abnormalities and several cardiovascular risk factors. The pharmacological actions of FGF21 make it attractive as future drug for treating diabetes. Indeed, FGF21 is already in clinical trials [14]. Clearly, a great amount of studies are needed to better elucidate the pathophysiological significance of FGF21 up-regulation in pregnancy when hyperglycemia, insulin resistance and dyslipidemia are present. Further studies will be required to investigate the pharmacological effects of FGF21 on impaired glucose metabolism and insulin resistance in pregnancy.

In the present study, we failed to find significant relationships between FGF19 or FGF21 and preconception BMI. Both findings are in contrast to several previous studies indicating that FGF19 levels correlated inversely with BMI [21] and FGF21 levels were positively associated with BMI or adiposity [22]. It is likely that the narrow range of BMI in our studied population precluded detection of such association in contrast with the extremely wide range of BMI values in previous studies. Additionally, we can not determine the body fat percentage only using BMI index. Thus the relationships between serum FGF19 or FGF21 levels and adiposity warrants further investigation.

Due to their similar receptor specificities and co-receptor requirements, FGF19 and FGF21 shared many common properties and have been thought to be interchangeable in metabolic regulation [15]. However, in our study, we showed serum FGF19 levels were reduced in GDM patients, in contrast with FGF21 levels. Furthermore, FGF19 levels were negatively associated with insulin resistance and positively associated with adiponectin, in opposite with FGF21. These results are in line with data from recent study by Gallego and coworkers in which both serum FGF19 and FGF21 levels were quantified in HIV-infected patients and healthy controls [33]. Here, serum FGF19 levels were reduced in HIV-infected patients with metabolic disturbances including hyperglycemia and insulin resistance, whereas FGF21 levels were increased. Moreover, one study showed that insulin stimulated FGF21 expression during hyperinsulinemic clamp in obese T2DM patients [23], while the other study demonstrated that acute hyperinsulinemia tended to decrease FGF19 levels in healthy and T2DM subjects [21]. All these findings indicate one piece of important information. Though FGF19 and FGF21 share many similarities in the actions on metabolism, the regulations of the two hormones in humans are different and partially manifested in circulating expressions. We speculate that both factors are regulated by insulin resistance, but the precise mechanism may be complicated. FGF19 is presumably released from the small intestine when bile acids traversing the enterocytes activate the intestinal bile acid receptor FXR. FGF21 mRNA is abundant in pancreas and testis, and also in the liver where it is induced by fasting and ketogenic diets under control of the peroxisome proliferator-activator receptor α (PPARα) [14]. Insulin resistance should have different effects on FXR and PPARα. That should be complicated and involved rather than simple or via a single signaling pathway. The precise mechanism remains to be further investigated. On the other hand, FGF19 and FGF21 levels may be differentially affected by feeding or nutritional status. A diurnal variation has been demonstrated for FGF19, with clear postprandial peaks that are abolished when food is omitted [34]. In animal studies, unlike FGF19, FGF21 expression is induced in various tissues in response to both fasting and feeding [14]. But FGF21 in humans may exhibit different effects and/or bioactivity relative to rodents. For example, the chronic malnutrition in humans is associated with markedly reduced circulating levels of FGF21 [41]. FGF19 and FGF21 expression may be differentially induced in response to feeding status in GDM patients. This need to be further studied.

In our study, we found that the spread in the FGF19 and FGF21 levels were quite large in all groups. There is a wide interindividual variation of fasting levels for FGF19 and, in particular, FGF21. Genetic variation in protein synthesis, processing, and elimination may be important for this variation, and this warrants further exploration [42]. Additionally, both FGF19 and FGF21 levels are at least partially dependent upon feeding or nutritional status. Circulating levels of FGF19 and FGF21 measured after overnight fasting seem to be relatively stable in an individual over time [42]. In our study, the blood samples taken for the analysis of FGF19 and FGF21 were taken between 08:00 and 09:00 AM after overnight fasting at the time of OGTT. Thus, we acquired the relatively stable levels of FGF19/FGF21 in all subjects. Here, we speculate that the spread of FGF19/FGF21 levels in groups are related to the genetic variation or insulin sensitivity.

There are several limitations in the current study. Based on our cross-sectional study design, reduced FGF19 level or increased FGF21 level can only be defined as a marker of GDM. Our study is preliminary as the number of patients was small and the results cannot be generalized beyond Chinese adults with GDM. Furthermore, the possible changes of local FGF19 and FGF21 concentrations in the liver, adipose and other tissues that are not reflected in the circulation may not have been noticed in this study.

In conclusion, we demonstrated that circulating levels of FGF19 were significantly reduced in patients with GDM relative to healthy pregnant subjects. Serum FGF19 levels were independently and negatively associated with insulin resistance and preconception PCOS history in both GDM and healthy pregnant women. In contrast, circulating levels of FGF21 were increased in patients with GDM as compared with healthy pregnant controls. Additionally, serum FGF21 levels independently and positively correlated with insulin resistance and serum triglycerides. Considering the different situation between FGF19 and FGF21 in the present and previous studies, we suggest that reduced serum FGF19 levels could be involved in the pathophysiology of GDM and GDM associated complications, while increased serum FGF21 levels could be in a compensatory response to this disease. Further studies are necessary to better elucidate the physiological significance of these findings. Considering the glucose- and insulin-lowering actions of FGF19 and/or FGF21 in diabetic rodents, the potential of FGF19 and/or FGF21 treatment to ameliorate impaired glucose tolerance and insulin resistance in GDM patients warrants future attention.

## References

[1] Zhu WW, Fan L, Yang HX, Kong LY, Su SP, et al (2013) Fasting Plasma Glucose at 24–28 Weeks to Screen for Gestational Diabetes Mellitus: New evidence from China. Diabetes Care 36: 2038–2040. 23536582 10.2337/dc12-2465 PMC3687275

[2] HAPO Study Cooperative Research Group (2009) Hyperglycemia and Adverse Pregnancy Outcome (HAPO) Study: associations with neonatal anthropometrics. Diabetes 58: 453–459. 19011170 10.2337/db08-1112 PMC2628620

[3] Barnes-Powell LL (2007) Infants of diabetic mothers: the effects of hyperglycemia on the fetus and neonate. Neonatal Netw 26: 283–290. 17926658 10.1891/0730-0832.26.5.283

[4] Bo S, Valpreda S, Menato G, Bardelli C, Botto C, et al (2007) Should we consider gestational diabetes a vascular risk factor? Atherosclerosis 194: e72–e79. 17055515 10.1016/j.atherosclerosis.2006.09.017

[5] Bellamy L, Casas JP, Hingorani AD, Williams D (2009) Type 2 diabetes mellitus after gestational diabetes: a systematic review and meta-analysis. Lancet 373: 1773–1779. 19465232 10.1016/S0140-6736(09)60731-5

[6] Damm P (2009) Future risk of diabetes in mother and child after gestational diabetes mellitus. Int J Gynaecol Obstet 104: S25–S26. 19150058 10.1016/j.ijgo.2008.11.025

[7] Bergmann K, Sypniewska G (2013) Diabetes as a complication of adipose tissue dysfunction. Is there a role for potential new biomarkers? Clin Chem Lab Med 51: 177–185. 23241684 10.1515/cclm-2012-0490

[8] Retnakaran R, Hanley AJ, Raif N, Hirning CR, Connelly PW, et al (2005) Adiponectin and beta cell dysfunction in gestational diabetes: pathophysiological implications. Diabetologia 48: 993–1001. 15778860 10.1007/s00125-005-1710-x

[9] Lain KY, Daftary AR, Ness RB, Roberts JM (2008) First trimester adipocytokine concentrations and risk of developing gestational diabetes later in pregnancy. Clin Endocrinol (Oxf)69: 407–411. 10.1111/j.1365-2265.2008.03198.x 18284645

[10] Qiu C, Williams MA, Vadachkoria S, Frederick IO, Luthy DA (2004) Increased maternal plasma leptin in early pregnancy and risk of gestational diabetes mellitus. Obstet Gynecol 103: 519–525. 14990416 10.1097/01.AOG.0000113621.53602.7a

[11] Lewandowski KC, Stojanovic N, Press M, Tuck SM, Szosland K, et al (2007) Elevated serum levels of visfatin in gestational diabetes: a comparative study across various degrees of glucose tolerance. Diabetologia 50: 1033–1037. 17334748 10.1007/s00125-007-0610-7

[12] Kirwan JP, Hauguel-De Mouzon S, Lepercq J, Challier JC, Huston-Presley L, et al (2002) TNF-alpha is a predictor of insulin resistance in human pregnancy. Diabetes 51: 2207–2213. 12086951 10.2337/diabetes.51.7.2207

[13] Krzyzanowska K, Zemany L, Krugluger W, Schernthaner GH, Mittermayer F, et al (2008) Serum concentrations of retinol-binding protein 4 in women with and without gestational diabetes. Diabetologia 51: 1115–1122. 18437353 10.1007/s00125-008-1009-9 PMC2676863

[14] Potthoff MJ, Kliewer SA, Mangelsdorf DJ (2012) Endocrine fibroblast growth factors15/19 and 21: from feast to famine. Genes Dev 26: 312–324. 22302876 10.1101/gad.184788.111 PMC3289879

[15] Adams AC, Coskun T, Rovira AR, Schneider MA, Raches DW, et al (2012) Fundamentals of FGF19 & FGF21 action in vitro and in vivo. PLoS One 7: e38438. 22675463 10.1371/journal.pone.0038438 PMC3365001

[16] Fu L, John LM, Adams SH, Yu XX, Tomlinson E, et al (2004) Fibroblast growth factor 19 increases metabolic rate and reverses dietary and leptin-deficient diabetes. Endocrinology 145: 2594–2603. 14976145 10.1210/en.2003-1671

[17] Fisher FM, Estall JL, Adams AC, Antonellis PJ, Bina HA, et al (2011) Integrated regulation of hepatic metabolism by fibroblast growth factor 21 (FGF21) in vivo. Endocrinology 152: 2996–3004. 21712364 10.1210/en.2011-0281 PMC3138239

[18] Kir S, Beddow SA, Samuel VT, Miller P, Previs SF, et al (2011) FGF19 as a postprandial, insulin-independent activator of hepatic protein and glycogen synthesis. Science 331: 1621–1624. 21436455 10.1126/science.1198363 PMC3076083

[19] Wente W, Efanov AM, Brenner M, Kharitonenkov A, Köster A, et al (2006) Fibroblast growth factor-21 improves pancreatic beta-cell function and survival by activation of extracellular signal-regulated kinase 1/2 and Akt signaling pathways. Diabetes 55: 2470–2478. 16936195 10.2337/db05-1435

[20] Barutcuoglu B, Basol G, Cakir Y, Cetinkalp S, Parildar Z, et al (2011) Fibroblast growth factor-19 levels in type 2 diabetic patients with metabolic syndrome. Ann Clin Lab Sci 41: 390–396. 22166511

[21] Mráz M, Lacinová Z, Kaválková P, Haluzíková D, Trachta P, et al (2011) Serum concentrations of fibroblast growth factor 19 in patients with obesity and type 2 diabetes mellitus: the influence of acute hyperinsulinemia, very-low calorie diet and PPAR-α agonist treatment. Physiol Res 60: 627–636. 21574752 10.33549/physiolres.932099

[22] Zhang X, Yeung DC, Karpisek M, Stejskal D, Zhou ZG, et al (2008) Serum FGF21 levels are increased in obesity and are independently associated with the metabolic syndrome in humans. Diabetes 57: 1246–1253. 18252893 10.2337/db07-1476

[23] Mraz M, Bartlova M, Lacinova Z, Michalsky D, Kasalicky M, et al (2009) Serum concentrations and tissue expression of a novel endocrine regulator fibroblast growth factor-21 in patients with type 2 diabetes and obesity. Clin Endocrinol (Oxf) 71: 369–375. 19702724 10.1111/j.1365-2265.2008.03502.x

[24] Chen C, Cheung BM, Tso AW, Wang Y, Law LS, et al (2011) High plasma level of fibroblast growth factor 21 is an Independent predictor of type 2 diabetes: a 5.4-year population-based prospective study in Chinese subjects. Diabetes Care 34: 2113–2115. 21750278 10.2337/dc11-0294 PMC3161267

[25] Bobbert T, Schwarz F, Fischer-Rosinsky A, Pfeiffer AF, Möhlig M, et al (2013) Fibroblast growth factor 21 predicts the metabolic syndrome and type 2 diabetes in Caucasians. Diabetes Care 36: 145–149. 22933429 10.2337/dc12-0703 PMC3526237

[26] Stein S, Stepan H, Kratzsch J, Verlohren M, Verlohren HJ, et al (2010) Serum fibroblast growth factor 21 levels in gestational diabetes mellitus in relation to insulin resistance and dyslipidemia. Metabolism 59: 33–37. 19699495 10.1016/j.metabol.2009.07.003

[27] Yang HX (2012) Diagnostic criteria for gestational diabetes mellitus (WS 331-2011). Chin Med J (Engl) 125: 1212–1213. 22613589

[28] Rotterdam ESHRE/ASRM-Sponsored PCOS consensus workshop group (2004) Revised 2003 consensus on diagnostic criteria and long-term health risks related to polycystic ovary syndrome (PCOS). Hum Reprod 19: 41–47. 14688154 10.1093/humrep/deh098

[29] Matthews DR, Hosker JP, Rudenski AS, Naylor BA, Treacher DF, et al (1985) Homeostasis model assessment: insulin resistance and beta-cell function from fasting plasma glucose and insulin concentrations in man. Diabetologia 28: 412–419. 3899825 10.1007/BF00280883

[30] Tomlinson E, Fu L, John L, Hultgren B, Huang X, et al (2002) Transgenic mice expressing human fibroblast growth factor-19 display increased metabolic rate and decreased adiposity. Endocrinology 143: 1741–1747. 11956156 10.1210/endo.143.5.8850

[31] Schreuder TC, Marsman HA, Lenicek M, van Werven JR, Nederveen AJ, et al (2010) The hepatic response to FGF19 is impaired in patients with nonalcoholic fatty liver disease and insulin resistance. Am J Physiol Gastrointest Liver Physiol 298: G440–G445. 20093562 10.1152/ajpgi.00322.2009

[32] Reiche M, Bachmann A, Lössner U, Blüher M, Stumvoll M, et al (2010) Fibroblast growth factor 19 serum levels: relation to renal function and metabolic parameters. Horm Metab Res 42: 178–181. 20013647 10.1055/s-0029-1243249

[33] Gallego-Escuredo JM, Domingo P, Gutiérrez Mdel M, Mateo MG, Cabeza MC, et al (2012) Reduced levels of serum FGF19 and impaired expression of receptors for endocrine FGFs in adipose tissue from HIV-infected patients. J Acquir Immune Defic Syndr 61: 527–534. 23187887 10.1097/QAI.0b013e318271c2c7

[34] Lundasen T, Gälman C, Angelin B, Rudling M (2006) Circulating intestinal fibroblast growth factor 19 has a pronounced diurnal variation and modulates hepatic bile acid synthesis in man. J Intern Med 260: 530–536. 17116003 10.1111/j.1365-2796.2006.01731.x

[35] Stejskal D, Karpísek M, Hanulová Z, Stejskal P (2008) Fibroblast growth factor-19: development, analytical characterization and clinical evaluation of a new ELISA test. Scand J Clin Lab Invest 68: 501–507. 18609104 10.1080/00365510701854967

[36] Shin DJ, Osborne TF (2009) FGF15/FGFR4 integrates growth factor signaling with hepatic bile acid metabolism and insulin action. J Biol Chem 284: 11110–11120. 19237543 10.1074/jbc.M808747200 PMC2670116

[37] Chang RJ, Nakamura RM, Judd HL, Kaplan SA (1983) Insulin resistance in nonobese patients with polycystic ovarian disease. J Clin Endocrinol Metab 57: 356–359. 6223044 10.1210/jcem-57-2-356

[38] Boomsma CM, Eijkemans MJ, Hughes EG, Visser GH, Fauser BC, et al (2006) A meta-analysis of pregnancy outcomes in women with polycystic ovary syndrome. Hum Reprod Update 12: 673–683. 16891296 10.1093/humupd/dml036

[39] Toulis KA, Goulis DG, Farmakiotis D, Georgopoulos NA, Katsikis I, et al (2009) Adiponectin levels in women with polycystic ovary syndrome: a systematic review and a meta-analysis. Hum Reprod Update 15: 297–307. 19261627 10.1093/humupd/dmp006

[40] Kharitonenkov A, Wroblewski VJ, Koester A, Chen YF, Clutinger CK, et al (2007) The metabolic state of diabetic monkeys is regulated by fibroblast growth factor-21. Endocrinology 148: 774–781. 17068132 10.1210/en.2006-1168

[41] Dostálová I, Kaválková P, Haluzíková D, Lacinová Z, Mráz M, et al (2008) Plasma concentrations of fibroblast growth factors 19 and 21 in patients with anorexia nervosa. J Clin Endocrinol Metab 93: 3627–3632. 18559909 10.1210/jc.2008-0746

[42] Angelin B, Larsson TE, Rudling M (2012) Circulating fibroblast growth factors as metabolic regulators–a critical appraisal. Cell Metab 16: 693–705. 23217254 10.1016/j.cmet.2012.11.001

